# Intranasal Delivery of Ketamine Induces Cortical Disinhibition

**DOI:** 10.1523/ENEURO.0107-23.2023

**Published:** 2024-01-16

**Authors:** Xin Qiao, Steven F. Grieco, Zhaoxia Yu, Todd C. Holmes, Xiangmin Xu

**Affiliations:** ^1^Department of Anatomy and Neurobiology, School of Medicine, University of California, Irvine 92697, California; ^2^Center for Neural Circuit Mapping, University of California, Irvine 92697, California; ^3^Department of Statistics, Donald Bren School of Information and Computer Sciences, Irvine 92697, California; ^4^Department of Physiology and Biophysics, School of Medicine, University of California, Irvine 92697, California

**Keywords:** depression, disinhibition, intranasal, ketamine, parvalbumin, visual cortex

## Abstract

Our previous studies find that subcutaneously administered (s.c.) subanesthetic ketamine promotes sustained cortical disinhibition and plasticity in adult mouse binocular visual cortex (bV1). We hypothesized that intranasal delivery (i.n.) of subanesthetic ketamine may have similar actions. To test this, we delivered ketamine (10 mg/kg, i.n.) to adult mice and then recorded excitatory pyramidal neurons or PV+ interneurons in L2/3 of bV1 slices. In pyramidal neurons the baseline IPSC amplitudes from mice treated with ketamine are significantly weaker than those in control mice. Acute bath application of neuregulin-1 (NRG1) to cortical slices increases these IPSC amplitudes in mice treated with ketamine but not in controls. In PV+ interneurons, the baseline EPSC amplitudes from mice treated with ketamine are significantly weaker than those in control mice. Acute bath application of NRG1 to cortical slices increases these EPSC amplitudes in mice treated with ketamine but not in controls. We also found that mice treated with ketamine exhibit increased pCREB staining in L2/3 of bV1. Together, our results show that a single intranasal delivery of ketamine reduces PV+ interneuron excitation and reduces pyramidal neuron inhibition and that these effects are acutely reversed by NRG1. These results are significant as they show that intranasal delivery of ketamine induces cortical disinhibition, which has implications for the treatment of psychiatric, neurologic, and ophthalmic disorders.

## Significance Statement

We show that intranasal delivery of a single subanesthetic dose of ketamine in the mouse model has profound effects on the microcircuitry in sensory cortex. We found that intranasal delivery of ketamine reduces parvalbumin-expressing interneuron (PV+) excitation and reduces pyramidal neuron inhibition. Disinhibition is a critical step for promoting cortical neural plasticity by ketamine therapy. Despite the very important clinical use of intranasal ketamine, surprisingly few publications have reported using this route of administration in animal models for translational research. Here, we show how intranasal ketamine modulates the circuitry to bring about cortical disinhibition.

## Introduction

Ketamine is a Schedule III controlled substance which has been used for decades as a dissociative anesthetic for the induction and maintenance of global anesthesia. It is a racemic mixture of two mirror enantiomers, *R*- and *S*-ketamine, and is available as ketamine hydrochloride. Much more recently, a single subanesthetic dose of ketamine has been found to improve psychiatric symptoms in adults with treatment-resistant depression (TRD), and these effects are sustained, lasting up to 2 weeks (Berman et al., 2000; Zarate et al., 2006; Price et al., 2009; Duman and Aghajanian, 2012). This progress led to the FDA approval of Spravato (*S*-ketamine) in 2019 for its use as a nasal spray for TRD in adults with major depressive disorder (MDD) and acute suicidal ideation or behavior, in conjunction with an oral antidepressant (Turner, 2019). As Spravato has several potential risks associated with its use clinically, it is subject to very strict safety controls and is administered under a program called a Risk Evaluation and Mitigation Strategy (REMS). Despite these risks, ketamine has been called “arguably the most important discovery in half a century” by some within the field of psychiatry (Duman and Aghajanian, 2012).

A single subanesthetic dose of ketamine rapidly improves psychiatric symptoms within minutes or hours, and this improvement can last for weeks, much longer than the presence of the drug or its metabolites in the patient. This sustained therapeutic effect of ketamine strongly suggests that it may work by promoting neural plasticity in the brain. As ketamine has been shown experimentally to rapidly promote synaptogenesis, it fits the profile of a “psychoplastogen,” a therapeutic that rapidly induces neural plasticity following a single dose leading to long-lasting changes in behavior (Grieco et al., 2022). Since ketamine is an *N*-methyl-D-aspartate receptor (NMDAR) antagonist and it also has been shown experimentally to rapidly induce cortical disinhibition (this has mostly been studied acutely; Homayoun and Moghaddam, 2007), the “disinhibition hypothesis” asserts that ketamine might promote neural plasticity by antagonizing NMDARs on GABAergic inhibitory neurons leading to cortical disinhibition (Homayoun and Moghaddam, 2007; Browne and Lucki, 2013; Krystal et al., 2013; Fan et al., 2018; Widman and McMahon, 2018; Ali et al., 2020). However, (1) it is not clear why ketamine would act on NMDARs exclusively (or predominantly) at inhibitory neurons, and (2) it has been shown (in 2016) that ketamine's antidepressant drug-like effects in animal models may not depend on NMDARs (Zanos et al., 2016). These findings sent the field searching for alternative mechanistic explanations of ketamine-mediated therapeutic effects.

At that time our laboratory made the un-related discovery that downregulation of neuregulin-1 (NRG1) expression in parvalbumin-expressing (PV+) interneurons results in cortical disinhibition and ocular dominance plasticity (ODP) during the juvenile critical period (CP) (Sun et al., 2016; Grieco et al., 2019). Upon searching the literature, we found that chronic higher doses of ketamine have been used in preclinical research to model schizophrenia (SCZ) and that this results in a loss of PV+ interneuron phenotype in the brain (Behrens et al., 2007; Li et al., 2010; Zanos et al., 2016). Based on this information, we hypothesized that a single subanesthetic dose of ketamine might promote ODP by reducing PV+ interneuron excitation. There was a precedent in the field for studying the plasticity-promoting mechanisms of antidepressants using ODP as a model, as Eero Castren's group in Finland has published extensively showing how Prozac (fluoxetine) promotes ODP in adult animals (Maya Vetencourt et al., 2008; Umemori et al., 2018). Upon testing our hypothesis, we found that subcutaneous (s.c.) administration of a single subanesthetic dose of ketamine downregulates NRG1 expression in PV+ interneurons, and this results in cortical disinhibition and ODP in adult bV1 (Grieco et al., 2020). This effect of ketamine on ODP in adult animals has since been corroborated by other groups (Casarotto et al., 2021; Cannarozzo et al., 2022).

Intranasal administration of ketamine is used in the clinic on human patients, so we decided to test herein if this route of delivery for a single subanesthetic dose of ketamine induces disinhibition in the cortex of the mouse. This is important to establish as we have previously observed disinhibition in bV1 after peripheral delivery of ketamine. We found that intranasal delivery (i.n.) of a single subanesthetic dose of ketamine reduces PV+ interneuron excitation and reduces pyramidal neuron inhibition and that these effects are reversed by acute bath application of NRG1 to cortical slices. These results are significant as they show that intranasal delivery of ketamine induces disinhibition in cortex, which has implications for the treatment of psychiatric, neurologic, and ophthalmic disorders.

## Materials and Methods

### Animals

All experimental procedures and protocols were approved by the Institutional Animal Care and Use Committee of UCI. To genetically label PV+ interneurons, we crossed PV-Cre mice with Ai9 reporter mice (Jax #007905). The animals (2–5 mice per cage; all males) at ages 6–8 weeks old were housed in a vivarium room with a 12 h light/dark cycle with access to food and water *ad libitum*. We had chosen to use males in this study because of a recently disproven assumption that female mice display more behavioral variability than male mice (Levy et al., 2023; Wiseman, 2023). The mice were randomly assigned to groups treated with either saline or subcutaneous ketamine or intranasal ketamine. A single dosage of ketamine (10 mg/kg, ketamine hydrochloride; Vedco) was used for all intranasal or subcutaneous treatments. For intranasal injections, ketamine (100 mg/ml) was diluted in sterile saline solution (Nova Tech). Graduated filter tips (TipOne 1-200 µl, USA Scientific) and micropipettors (Labpipette, Labnet International) were used to apply 20 µl of the ketamine solution to each nostril, which is visually observed to be absorbed into the nostril by the mouse while being scruffed.

### Electrophysiology

Coronal sections (300 µm thick) of the visual cortex were cut from mouse with a vibratome (VT1200S, Leica Biosystems) in sucrose-containing artificial cerebrospinal fluid (ACSF; in mM, 85 NaCl, 75 sucrose, 2.5 KCl, 25 glucose, 1.25 NaH_2_PO_4_, 4 MgCl_2_, 0.5 CaCl_2_, and 24 NaHCO_3_). Slices were incubated for at least 1 h in standard ACSF (in mM, 126 NaCl, 2.5 KCl, 26 NaHCO_3_, 2 CaCl_2_, 2 MgCl_2_, 1.25 NaH_2_PO_4_, and 10 glucose) at 32°C before being transferred into slice-recording chambers. Throughout the cutting, incubation and recording, the solutions were continuously supplied with 95% O_2_ to 5% CO_2_.

We have previously described our methods for electrophysiological recording in detail, including the definitions of all reported parameters (Shi et al., 2010; Xu et al., 2010). For our more recent publications, we used these same methods (Grieco et al., 2020, 2021). Briefly, whole-cell recordings were performed in oxygenated ACSF at room temperature under a differential interference contrast/fluorescent Olympus microscope (BX51WI). Oxygenated ACSF was fed into the slice-recording chamber through a custom-designed flow system, driven by pressurized 95% O_2_ to 5% CO_2_ (3 PSI) with a perfusion flow rate of ∼2 ml/min. Slices were first carefully examined under a 4× objective for targeting either L2/3 pyramidal cells or PV+ interneurons that express red fluorescent protein tdTomato within the visual cortex. To perform whole-cell recordings, neurons were visualized at high magnification (60× objective, 0.9 NA; LUMPlanFl/IR, Olympus America). The cell bodies of recorded neurons were at least 50 µm below the surface of the slice. To record inhibitory postsynaptic currents (IPSCs) in pyramidal cells, patch pipettes (4–6 MΩ resistance) made of borosilicate glass were filled with a cesium-based internal solution containing (in mM) 130 CsOH, 130 D-gluconic acid, 0.2 EGTA, 2 MgCl_2_, 6 CsCl, 10 HEPES, 2.5 ATP-Na, 0.5 GTP-Na, and 10 phosphocreatine, pH 7.2 (300 mOsm). The membrane potential of the pyramidal cell was held at 10 mV. Electrically evoked IPSCs in L2/3 were recorded by preferentially activating L4 to L2/3 feedforward projections to L2/3 PYR interneurons and the electrical stimulation electrode was placed in L4. The stimulator was placed in L4, 125 μm under the recorded cell in L2/3. Almost all cells respond to the 20 μA stimulus. A concentric bipolar electrode (CBAPB75, FHC) and a stimulus isolator (A365, World Precision Instruments) were used. The inter-stimulus interval is 1 min.

Electrically evoked excitatory postsynaptic currents (EPSCs) in L2/3 were recorded by preferentially activating L4 to L2/3 feedforward projections to L2/3 PV+ interneurons, and the electrical stimulation electrode was placed in L4. An internal solution containing (in mM) 126 K-gluconate, 4 KCl, 10 HEPES, 4 ATP-Mg, 0.3 GTP-Na, and 10 phosphocreatine, pH 7.2 (300 mOsm), was applied. The membrane potential of PV+ interneurons was held at −70 mV. Electrically evoked EPSCs in PV+ interneurons were stimulated by an electrical stimulation in L4 through L4 to L2/3 feedforward projections. The patch pipettes also contained 0.1% biocytin for *post hoc* cell labeling and further morphological labeling. Only stable whole-cell recordings with good access resistance (<30 MΩ) were selected. Electrophysiological data were acquired with a MultiClamp 700B amplifier (Molecular Devices), data acquisition boards (models PCI MIO 16E-4 and 6713, National Instruments), and a custom-modified version of Ephus software. Data were digitized at 10 kHz. At all three time points (baseline, NRG1 application 20 min, washout 20 min), three trials were used. We measured the peak of each trial and then averaged the trials.

The slice experiments were not done blind to treatment condition, but analysis was performed blind. Approximately 1–2 cells were recorded from each animal. No data were excluded, thus alleviating concerns, except if the access resistance changed by >20% during the course of the experiment, indicating that the patch was inadequate.

### Immunohistochemistry

For immunohistochemical staining experiments, mice were first deeply anesthetized with Euthasol (sodium pentobarbital, 100 mg/kg, i.p.) and were then perfused transcardially with 5 ml of 1× phosphate-buffered saline (PBS, pH 7.3–7.4), followed by 20 ml 1× PBS containing 4% paraformaldehyde (PFA) and phosphatase inhibitor (PhosSTOP, 1 tablet for 20 ml, Roche). Brains were removed and maintained in 4% PFA for 24 h and then transferred to 30% sucrose in 1× PBS for 24 h. Then, using a freezing microtome (Leica SM2010R), coronal sections of the brain were taken at a 30 μm thickness. Mouse V1 coronal sections from the bregma −3.40 to −3.80 mm were used for immunohistochemical staining and analysis.

Free floating sections (2–3/well) were rinsed five times with 1× PBS and incubated in a blocking solution for 1 h at room temperature on a shaker. The blocking solution contained 5% normal donkey serum and 0.25% Triton X in 1× PBS. Sections were then incubated with the primary antibody diluted in blocking solution for 48 h at 4°C. After incubation with the primary antibody, brain sections were rinsed thoroughly with 1× PBS three times for 10 min each time and then incubated with the secondary antibody diluted in blocking solution for 2 h at room temperature. After the secondary antibody was rinsed off using 1× PBS three times for 10 min each time, sections were counterstained with 10 μM 4′,6-diamidino-2-phenylindole (Sigma-Aldrich) for 5 min to help distinguish cortical laminar structure and neuronal nuclei. Lastly, sections were rinsed and then mounted on microscope slides. Sections were coverslipped with Vectashield mounting medium (H-1000, Vector). To identify cAMP response element-binding protein (pCREB)-positive neurons, we used the primary rabbit anti-pCREB antibody (87G3; Cell Signaling; RRID: AB_2561044; 1:1,000) and an Alexa488-conjugated donkey anti-rabbit antibody (Jackson ImmunoResearch Laboratories).

Immunostained sections were examined, and 10× and 20× image stacks were acquired using a confocal microscope (FLUOVIEW Olympus FV3000 confocal laser scanning microscope). Image tiles, overlaying, maximum projections, and subset *z*-stack selections were performed using the Olympus image processing software (FV31S-acquisition). For fluorescent imaging, all sections of a staining series were acquired using the same settings (laser power, pinhole size, line scans), and data images were digitally processed identically.

Cell fluorescence intensity measurements were conducted by using Adobe Photoshop software (CS4 extended version, Adobe Systems). First, the background reading of the fluorescence level needs to be determined for each stained V1 section image. A total of 20–30 background squares (24 pixels × 24 pixels) are picked up at the image location that did not have any staining signals and record and average the mean gray value for all the background squares. This number represents the background mean gray value per pixel (defined as GB). Next, we record the cell area of L2/3 cells (number of pixels, defined as *A*) and the integrated density (whole-cell fluorescence intensity, defined as Dint) for each cell. Finally, a background subtraction method needs to be used for calculating the final corrected total cell fluorescence (CTCF). In our case, for each cell, CTCF = Dint − GB * *A*. We average the CTCF for each sample and followed with statistical analysis.

### Behavioral tests

PV-cre;Ai9 mice were habituated in the behavior room for 1 h before testing. Then they were subcutaneously or intranasally administered saline or ketamine at a dose of 10 mg/kg and individually placed in a rectangular open-field arena containing bedding (28 cm long and 23 cm wide). Mice were videotaped for 1 h (Logitech webcam software). Distance traveled and freezing behavior were analyzed with an open-source software package ezTrack (Pennington et al., 2019). Mice were perfused for immunohistochemical staining at 24 h after drug administration.

### Statistical analysis

The data presented in the present study were collected using different experimental designs, and data were plotted and tested statistically using GraphPad software. Several appropriate statistical tests were applied in the data analysis. These included ANOVA, *t* test, Kruskal–Wallis test, and Mann–Whitney *U* test. When normality does not hold or sample sizes are small, parametric results might not be accurate. Normality was tested with a Kolmogorov–Smirnov test. For some results reported in the paper, we thus use nonparametric tests. We substituted Kruskal–Wallis test for one-way ANOVA and Mann–Whitney *U* test for two-sample *t* test, for example. We also performed power analysis using Cohen's *D* to attain effect size.

## Results

### Sustained cortical disinhibition of pyramidal neurons evoked by intranasal delivery of subanesthetic ketamine

**Figure 1.**
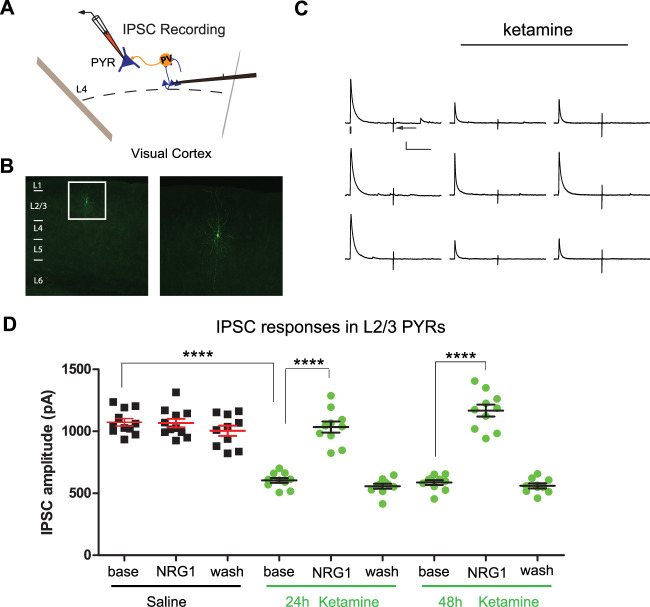
Intranasal delivery of a single subanesthetic dose of ketamine evokes sustained cortical disinhibition, which is reversed by exogenous NRG1. ***A***, ***B***, Schematic of recording electrically evoked IPSCs in L2/3 pyramidal (PYR) neurons of bV1 by preferentially activating L4→L2/3 feedforward projections to L2/3 PV+ interneurons through L4 electrical stimulation. Recorded neurons are filled with biocytin for *post hoc* verification. ***C***, ***D***, Electrically evoked IPSCs in L2/3 PYRs of bV1 from mice with intranasal delivery of a single subanesthetic dose of ketamine (10 mg/kg, i.n.; 24 and 48 h). For each neuron, the recording is performed through baseline, NRG1 application, and washout. Recordings are from different neurons and different mice at time points that are on different days (24 h and 48 h). ***C***, Representative example traces of IPSCs maintained on the same neurons at baseline, after NRG1 application, and after washout under different treatment conditions. For each trial, electrical stimulation (2 ms, 20 µA) is applied, represented by a black tick beneath one example trace. The arrow indicates the current injection response to monitor access resistance during the experiment. Intranasal ketamine reduces evoked IPSCs to PYR neurons at 24 h and 48 h after ketamine treatment. Ketamine-evoked decreases in IPSCs are reversed with bath NRG1. Acute bath application of NRG1 (5 nM) increases inhibitory input to L2/3 PYRs within 10–20 min, and this is washed out within 20 min. Traces show IPSCs before (top), during NRG1 application (20 min after NRG1 application; middle), and after washout (bottom). In control mice (left column), NRG1 does not affect IPSCs. In mice that received intranasal delivery of ketamine, IPSC amplitudes at baseline are significantly reduced compared with controls for both the 24 and 48 h time points. These reduced inhibitory inputs are increased by NRG1 application (middle row) and this increase can be washed out (bottom row). ***D***, Summary data of IPSC amplitudes at baseline under different treatment conditions (control, 24, and 48 h after intranasal ketamine treatment). Compared with those in control, IPSC amplitudes are significantly reduced after ketamine treatment [control: *n* = 11 cells (10 cells have washout data) from 10 slices of 5 mice; 24 h: *n* = 10 cells (all cells have washout data) from 10 slices of 4 mice; 48 h: *n* = 10 cells (9 cells have washout data) from 10 slices of 4 mice (two-way ANOVA with *post hoc* Tukey’s HSD: ketamine vs control, 24 h *p* < 0.0001; 48 h *p* < 0.0001)] and NRG1 vs baseline comparison at 24 and 48 h (two-way ANOVA with *post hoc* Tukey’s HSD: ketamine baseline vs NRG1: 24 h *p* < 0.0001, 48 h *p* < 0.0001).

Cortical disinhibition mediated by interneurons is an essential aspect of neural plasticity in the cortex (Kuhlman et al., 2013; Reh et al., 2020). Here, we determine if inhibition of pyramidal cells in bV1 is changed after intranasal delivery of ketamine (10 mg/kg; i.n.). To test this question, we performed whole-cell recording of electrically evoked IPSCs in pyramidal cells in layer 2/3 (L2/3) of bV1 by activating L4→L2/3 feedforward projections to L2/3 interneurons through L4 electrical stimulation (Fig. 1*A*; Sun et al., 2016; Grieco et al., 2020, 2021). We examined evoked inhibition of L2/3 pyramidal cells 24 and 48 h after intranasal delivery of ketamine (or saline). We found that after intranasal delivery of ketamine, excitatory pyramidal neuron inhibition is reduced at both the 24 and 48 h time points. Baseline IPSC amplitudes from ketamine-treated mice are significantly weaker than those in control mice [Fig. *1C*,*D*; control: 1073 ± 30.04 pA, *n* = 11 cells; 24 h: 603.8 ± 19.63 pA, *n* = 10 cells; 48 h: 586.4 ± 19.33 pA, *n* = 10 cells (two-way ANOVA with *post hoc* Tukey’s HSD: ketamine vs baseline, 24 h *p* < 0.0001; 48 h *p* < 0.0001)]. We then tested whether NRG1 increases pyramidal cell inhibition after intranasal delivery of ketamine. The recombinant NRG1 that we apply to the bath of cortical slices contains the epidermal growth factor core domain of NRG1-β1. We found that the cortical slice bath application of NRG1 increases IPSC amplitudes in pyramidal neurons from mice that had intranasal delivery of ketamine and that NRG1 reverses the reductions in inhibition in excitatory pyramidal cells that is observed after ketamine treatment [Fig. 1*C*,*D*; baseline, NRG1, and washout with saline: baseline = 1073.5 ± 30.0, *n* = 11 cells; NRG1 = 1066.3 ± 34.4, *n* = 11 cells; and with washout = 1004.4 ± 41.0, *n* = 10 cells, mean ± SEM; baseline, NRG1, and washout at 24 h KET: baseline = 603.8 ± 19.6, *n* = 10 cells; NRG1 = 1034.3 ± 44.8, *n* = 10 cells; and with washout = 556.5 ± 19.9, *n* = 10 cells, mean ± SEM; baseline, NRG1, and washout at 48 h KET: baseline = 586.5 ± 19.3, *n* = 10 cells; NRG1 = 1167.4 ± 48.5, *n* = 10 cells; and with washout = 560.1 ± 20.2, *n* = 10 cells, mean ± SEM (two-way ANOVA with *post hoc* Tukey’s HSD: baselines vs NRG1s, control *p* > 0.9998; 24 h *p* < 0.0001; 48 h *p* < 0.0001)]. NRG1 has no effects on IPSC amplitudes in pyramidal neurons from control animals. The effect of NRG1 application to brain slices of ketamine-treated animals is acute as IPSC amplitudes return to baseline levels after NRG1 washout (Fig. 1*D*). Together, these results support that a single intranasal delivery of a subanesthetic dose of ketamine decreases inhibition of pyramidal cells in bV1 and that this process is acutely reversible by NRG1 signaling.

### Sustained loss of PV+ interneuron excitation by intranasal delivery of subanesthetic ketamine

**Figure 2.**
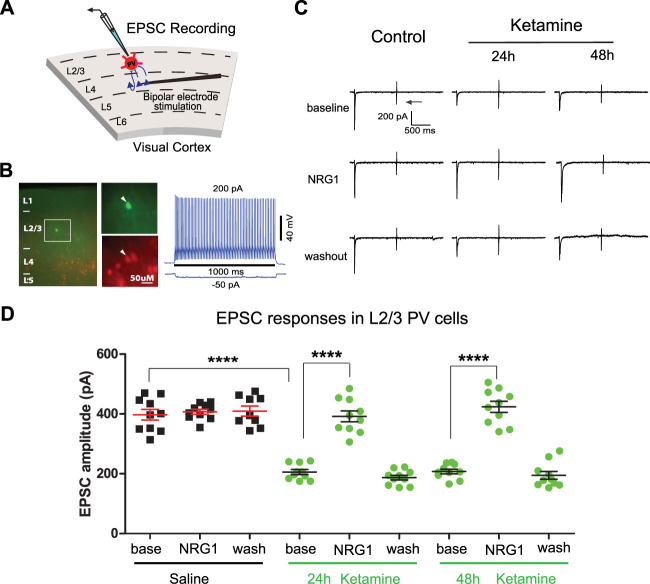
Intranasal delivery of a single subanesthetic dose of ketamine evokes sustained inhibition of PV+ interneurons, which is reversed by exogenous NRG1. ***A***, ***B***, Schematic of electrically evoked EPSCs in L2/3 PV+ interneurons of bV1 by activating L4→L2/3 projecting excitatory neurons through L4 electrical stimulation. Recorded neurons from PV-Cre;Ai9 mice are filled with biocytin for *post hoc* verification. ***C–E***, Electrically evoked EPSCs in L2/3 PV+ interneurons of bV1 from mice with intranasal delivery of a single subanesthetic dose of ketamine (10 mg/kg, i.n.; 24 and 48 h). For each neuron, the recording is performed through baseline, NRG1 application, and washout. Recordings are from different neurons and different mice at time points that are on different days (24 h and 48 h). ***C***, Example EPSC traces maintained on the same neurons recorded at baseline, after NRG1 application, and after washout under different treatments. For each trial, electrical stimulation (2 ms, 20 µA) is applied. The arrow indicates the current injection response to monitor access resistance during the experiment. Intranasal ketamine reduces evoked EPSCs in PV+ interneurons at 24 h and 48 h after ketamine treatment. Ketamine-evoked decreases in EPSCs are reversed with NRG1. Acute bath application of NRG1 (5 nM) increases excitatory input to L2/3 PV+ interneurons within 10–20 min, and this is washed out within 20 min. Traces show EPSCs before (top), during NRG1 application (20 min after NRG1 application; middle), and after washout (bottom). In control mice (left column), NRG1 does not affect EPSCs. In mice that received intranasal delivery of ketamine, EPSC amplitudes at baseline are significantly reduced compared with controls for both the 24 and 48 h time points. Reduced excitatory input is increased by NRG1 application (middle row) and this increase can be washed out (bottom row). ***D***, Summary data of EPSC amplitudes at baseline under the different treatments (control, 24 and 48 h after intranasal ketamine treatment). Compared with those in control, EPSC amplitudes are significantly reduced after ketamine treatment [control: *n* = 10 cells (9 cells have washout data) from 10 slices of 4 mice; 24 h: *n* = 10 cells (all cells have washout data) from 10 slices of 4 mice; 48 h: *n* = 10 cells (all cells have washout data) from 10 slices of 4 mice; two-way ANOVA with *post hoc* Tukey’s HSD: ketamine vs control, 24 h *p* < 0.0001; 48 h *p* = 0.0001] and NRG1 vs baseline comparison at 24 and 48 h (two-way ANOVA with *post hoc* Tukey’s HSD: ketamine baseline vs NRG1, 24 h *p* < 0.0001, and 48 h *p* < 0.0001).

We aimed to determine if a reduction of PV+ interneuron excitation is implicated in the decreases in cortical inhibition induced by intranasal delivery of a single dose of subanesthetic ketamine. To test this, we performed whole-cell recording of electrically evoked EPSCs in PV+ interneurons (identified using PV-Cre;Ai9 mice) in L2/3 of bV1 by activating L4 with electrical stimulation 24 and 48 h after intranasal delivery of a single subanesthetic dose of ketamine (Fig. 2*A*). We found that after intranasal delivery of ketamine, PV+ interneuron excitation is reduced at both the 24 and 48 h time points. Baseline EPSC amplitudes from ketamine-treated mice are significantly weaker than those in control mice [Fig. 2*C*,*D*; control, 397.4 ± 17.73 pA; *n* = 10 cells; 24 h, 205.8 ± 8.37 pA; *n* = 10 cells; 48 h, 207.6 ± 7.76 pA; *n* = 10 cells (two-way ANOVA with *post hoc* Tukey’s HSD, ketamines vs control, 24 h *p* < 0.0001; 48 h *p* < 0.0001)]. We then tested whether NRG1 increases PV+ interneuron excitation after intranasal delivery of ketamine. We found that cortical slice bath application of NRG1 increases EPSC amplitudes in PV+ interneurons from mice that received intranasal delivery of ketamine, and thus NRG1 reverses the reductions in PV+ interneuron excitation that is observed after ketamine treatment [Fig. 2*C*,*D*; baseline, NRG1, and washout with saline: baseline = 397.4 ± 17.7, *n* = 10 cells; NRG1 = 407.3 ± 8.5, *n* = 10 cells; and with washout = 409.4 ± 16.8, *n* = 9 cells, mean ± SEM; baseline, NRG1, and washout at 24 h KET: baseline = 205.8 ± 8.4, *n* = 10 cells; NRG1 = 392.0 ± 18.1, *n* = 10 cells; and with washout = 187.0 ± 7.8, *n* = 10 cells, mean ± SEM; baseline, NRG1, and washout at 48 h KET: baseline = 207.6 ± 7.8, *n* = 10 cells; NRG1 = 423.9 ± 18.4, *n* = 10 cells; and with washout = 194.7 ± 13.2, *n* = 10 cells, mean ± SEM (two-way ANOVA with *post hoc* Tukey’s HSD: baselines vs NRG1s, control *p* > 0.9999; 24 h *p* < 0.0001; 48 h *p* < 0.0001)]. NRG1 has no effects on EPSC amplitudes in PV+ interneurons from control animals. The effect of NRG1 application to brain slices of ketamine-treated animals is acute as EPSC amplitudes return to baseline levels after NRG1 washout (Fig. 2*D*). These results support that a single intranasal delivery of a subanesthetic dose of ketamine decreases L2/3 PV+ interneuron excitation in bV1 and that this process is acutely reversible by NRG1 signaling.

### Sustained cortical disinhibition by intranasal or subcutaneous delivery of subanesthetic ketamine

**Figure 3.**
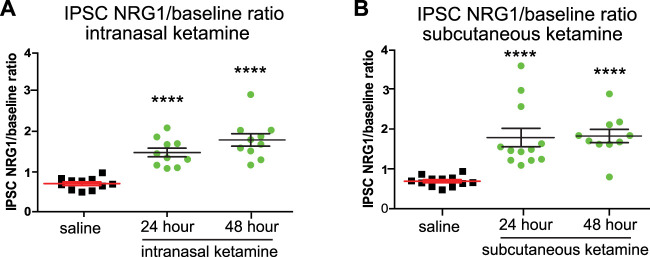
Both subcutaneous and intranasal delivery of ketamine evoke disinhibition of PYRs in L2/3 of bV1, which is reversed by NRG1. ***A***, ***B***, There is similarity of NRG1 effects on IPSCs in PYRs (NRG1/baseline ratio) for intranasal delivery of a single subanesthetic dose of ketamine (one-way ANOVA with *post hoc* Tukey’s HSD: NRG1/baseline ratio compared with control, 24 h *p* = 6.747 × 10^−5^, and at 48 h *p* = 2.904 × 10^−7^) and for subcutaneous delivery of a single subanesthetic dose of ketamine (Kruskal–Wallis test with *post hoc* Dunn's test: NRG1/baseline ratio compared with control, 24 h *p* = 9.55 × 10^−5^, and at 48 h *p* = 6.21 × 10^−5^).

We found that acute bath application of NRG1 to cortical slices increases excitatory pyramidal neuron inhibition and increases PV+ interneuron excitation in L2/3 of bV1 after intranasal delivery of single dose of subanesthetic ketamine (Figs. 1, 2). These effects of NRG1 are not observed in control animals that are administered intranasal saline. We then decided to compare the effects of NRG1 cortical slice bath application (IPSC amplitudes in excitatory pyramidal neurons; NRG1/baseline ratios) after a single subcutaneous delivery of subanesthetic ketamine (data replotted from Grieco et al., 2020) versus a single intranasal delivery of subanesthetic ketamine (data replotted from Fig. 1 herein). We found that acute bath application of NRG1 to cortical slices after either intranasal or subcutaneous delivery of ketamine results in an increase in IPSC amplitudes in excitatory pyramidal neurons [Fig. 3*A*,*B*; intranasal ketamine: NRG1/baseline ratio at 24 h, 1.73 ± 0.1, *n* = 10 cells; and at 48 h, 2.03 ± 0.15, *n* = 10 cells, mean ± SEM (one-way ANOVA with *post hoc* Tukey’s HSD: NRG1/baseline ratio compared with control, 24 h *p* = 6.747 × 10^−5^, and at 48 h *p* = 2.904 × 10^−7^); subcutaneous ketamine: NRG1/baseline ratio at 24 h, 1.97 ± 0.22, *n* = 12 cells; and at 48 h, 2.01 ± 0.16, *n* = 10 cells, mean ± SEM (Kruskal–Wallis test with *post hoc* Dunn's test: NRG1/baseline ratio compared with control, 24 h *p* = 9.55 × 10^−5^, and at 48 h *p* = 6.21 × 10^−5^)]. These results support that both routes of administration (subcutaneous or intranasal) of a single subanesthetic dose of ketamine achieve similar effects on cortical disinhibition of excitatory neurons in L2/3 bV1.

### Increased cortical pCREB by intranasal or subcutaneous delivery of subanesthetic ketamine

**Figure 4.**
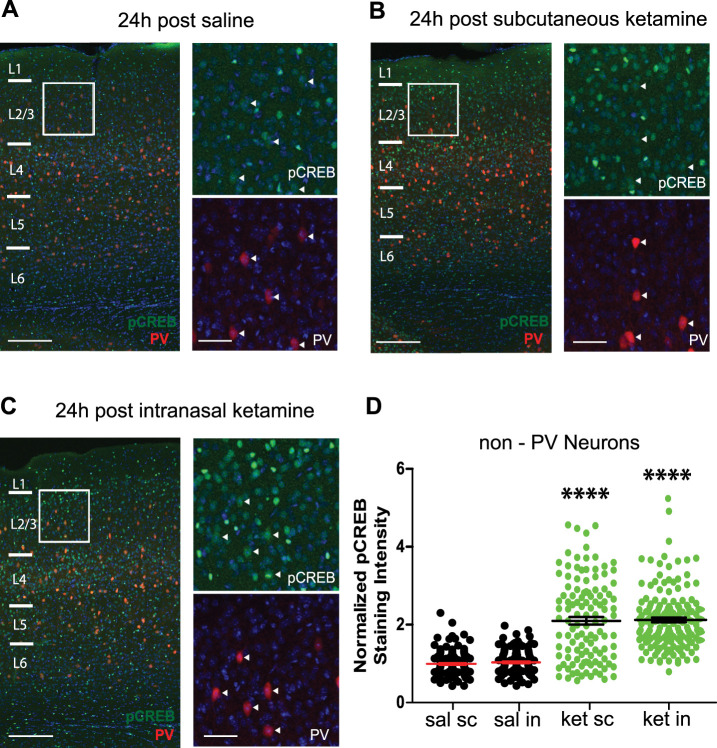
Intranasal delivery of ketamine increases pCREB in excitatory neurons. ***A***, Left, Confocal images of genetically labeled PV+ interneurons (red), pCREB immunostaining (green), and their overlay in bV1 in 2-month-old PV-Cre;Ai9 mice treated with saline; scale bar, 200 µm. The white box indicates the area of bV1 digitally enlarged (***A***, right). Right; scale bar, 50 µm. White arrowheads indicate that PV+ interneurons have very low pCREB. ***B***, ***C***, Confocal images of genetically labeled PV+ interneurons (red), pCREB immunostaining (green) and their overlay in bV1 in 2-month-old PV-Cre;Ai9 mice 24 h after a single subcutaneous (***B***) or intranasal (***C***) delivery of subanesthetic ketamine, respectively (10 mg/kg; s.c. or i.n.). ***D***, Quantification of the increase in pCREB immunoreactivity of non-PV+ interneurons, putative excitatory neurons in L2/3 of bV1 at 24 h after ketamine treatment [Kruskal–Wallis test: overall *p* < 0.00001, with *post hoc* Dunn's test (adjusted for multiple comparisons): subcutaneous ketamine vs saline *p* < 0.00001, intranasal ketamine vs saline *p* < 0.00001, mean ± SEM]. The overall normalized values (all groups normalized such that the mean of the combined sal group is equal to 1) of putative excitatory neurons pooled from different mice were grouped in accordance with different treatments (*n* = 9 mice with 220 cells for saline groups, *n* = 3 mice and 120 cells for subcutaneous ketamine, and *n* = 4 mice and 120 cells for intranasal ketamine).

Phosphorylated CREB is a molecular correlate of neural activity. As CREB phosphorylation at Ser133 is correlated with increased neural activity in excitatory neurons (Cohen and Greenberg, 2008), measuring this effect can provide further support for the induction of cortical disinhibition by ketamine. We found here that after intranasal delivery of a single subanesthetic dose of ketamine, pCREB immunostaining is increased in L2/3 of bV1 at the 24 h time point [Fig. 4; saline, 1 ± 0.03; subcutaneous ketamine, 2.1 ± 0.1; intranasal ketamine, 2.12 ± 0.06 (Kruskal–Wallis test with *post hoc* Dunn's test: saline vs subcutaneous ketamine, *p* < 0.00001; and saline vs intranasal ketamine, *p* < 0.00001); we combined saline groups for statistical comparisons; please see Table 1 for numbers of mice, slices, and cells]. Mice treated with saline or ketamine (s.c. and i.n.) are from the same cohort. These results support that intranasal delivery of a single subanesthetic dose of ketamine activates CREB by phosphorylation in L2/3 of bV1, suggesting the induction of cortical disinhibition and pCREB downstream signaling pathways involved in promoting neural plasticity (Cohen and Greenberg, 2008).

**Table 1.** Numbers of mice, slices, and cells for pCREB immunostaining of bV1 L2/3 at the 24 h time point after either subcutaneous or intranasal administration of ketamine

Mouse #	Averaged CTCF per mouse	Number of slices	Number of cells
Subcutaneous saline			
Mouse 1	4,279.90 ± 296.00	2	*N* = 40
Mouse 2	6,787.21 ± 368.34	2	*N* = 40
Mouse 3	6,068.20 ± 295.05	2	*N* = 40
Intranasal saline			
Mouse 1	5,734.64 ± 278.95	2	*N* = 40
Mouse 2	7,408.55 ± 314.74	2	*N* = 40
Mouse 3	3,553.15 ± 220.02	1	*N* = 20
Subcutaneous ketamine			
Mouse 1	9,977.01 ± 848.55	2	*N* = 40
Mouse 2	10,440.35 ± 958.19	2	*N* = 40
Mouse 3	16,292.58 ± 788.86	2	*N* = 40
Intranasal ketamine			
Mouse 1	10,796.62 ± 475.20	2	*N* = 40
Mouse 2	12,963.79 ± 702.20	2	*N* = 40
Mouse 3	14,399.56 ± 870.46	2	*N* = 40
Mouse 4	11,286.86 ± 505.04	2	*N* = 40

The average corrected total cell fluorescence (CTCF) is shown for each mouse.

### Intranasal delivery of subanesthetic ketamine affects behavioral activity

We hypothesized that there could be a difference in behavioral activity depending on the administration route of a single subanesthetic dose of ketamine, as a peripheral delivery route (subcutaneous) could potentially directly affect neuromuscular tissue, and a CNS-targeted delivery route (intranasal) could potentially directly affect the brain regions regulating behavioral activity (Nance et al., 2022). Others have reported that intranasal administration of single subanesthetic dose of ketamine reduces behavioral activity in an open arena, but this was not directly compared with subcutaneous administration (Chang et al., 2019; Goswami et al., 2021). To compare these delivery routes directly, we subcutaneously or intranasally delivered a single dose of subanesthetic ketamine to mice and then recorded their behavior. Mice were first habituated to the behavioral room for 1 h, then treated with drug, and then individually placed in a rectangular open field arena containing bedding (28 cm long and 23 cm wide) for 1 h—mice were place in the open arena 24 h later as well for 1 h. During the 1 h recording sessions, distance traveled and motionless epoch data were collected which were then analyzed with an open-source software package ezTrack (Pennington et al., 2019).

Two-way ANOVA analysis shows no significant interaction between the drug treatment (saline or ketamine) and the route of administration (subcutaneous or intranasal) for either distance traveled or motionless epochs at the 1 or 24 h time point [two-way ANOVA: distance (1 h), *p* = 0.5448; distance (24 h), *p* = 0.8582; freezing (1 h), *p* = 0.6461; freezing (24 h), *p* = 0.9192; please see Table 2 for a power analysis of this behavioral experiment]. Thus, there is not a significant difference in the route of drug administration on our behavioral measures—we therefore proceed using a two-way ANOVA without interactions analyses.

**Table 2.** Power analysis of behavioral experiments

	Subcutaneous vs intranasal ketamine			
	1 h dis	24 h dis	1 h freezing	24 h freezing
Cohen's *D*	0.78	0.32	0.25	0.08
Change (%)	17.01	−3.59	11.84	−4.67
Power (*n* = 8)	0.31	0.09	0.08	0.05
*n* (power = 80%)	27.00	150.00	253.00	2,302.00

The first row titled Cohen's *D* is the effect size for each comparison. The second row is the change in percentage. The third row is the power with the Cohen's *D* in the first row for *n* = 8 per group. The last row is the sample size per group required to reach 80% power.

**Figure 5.**
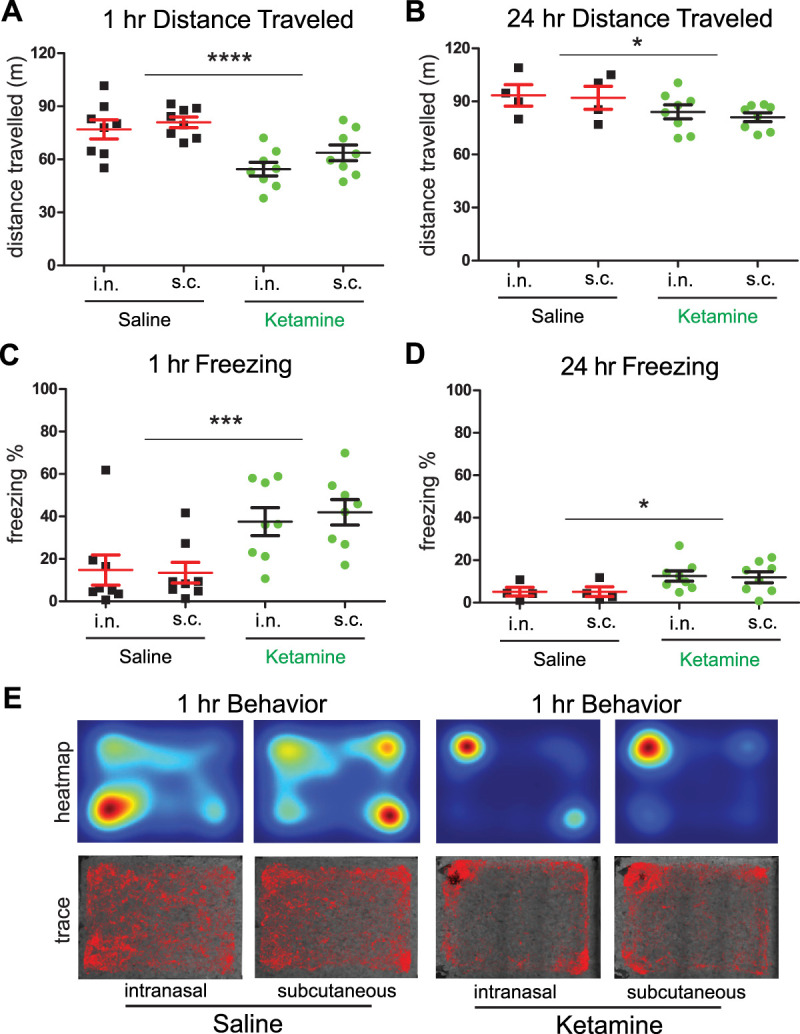
Delivery of subanesthetic ketamine affects behavioral activity. ***A***, ***B***, Summary data of distance traveled by mice during a 1 h epoch, at 1 h or 24 h after intranasal or subcutaneous delivery of a single subanesthetic dose of ketamine compared with saline treatment [distance traveled at 1 h: saline (i.n.), *n* = 8; ketamine (i.n.), *n* = 8; saline (s.c), *n* = 8; ketamine (s.c), *n* = 8 (two-way ANOVA: saline vs ketamine, *p* = 5.814 × 10^−5^; intranasal vs subcutaneous, *p* = 0.1261); distance traveled at 24 h: saline (i.n.), *n* = 4; ketamine (i.n.), *n* = 8; saline (s.c), *n* = 4; ketamine (s.c), *n* = 8 (two-way ANOVA: saline vs ketamine, *p* = 0.03112; intranasal vs subcutaneous, *p* = 0.5562); mean ± SEM]. ***C***, ***D***, Summary data of motionless epochs by mice during the 1 h experiment at 1 h or 24 h after intranasal or subcutaneous delivery of ketamine compared with saline treatment [distance traveled at 1 h: saline (i.n.), *n* = 8; ketamine (i.n.), *n* = 8; saline (s.c), *n* = 8; ketamine (s.c), *n* = 8 (two-way ANOVA: saline vs ketamine, *p* = 2.351 × 10^−4^; intranasal vs subcutaneous, *p* = 0.7992); distance traveled at 24 h: saline (i.n.), *n* = 4; ketamine (i.n.), *n* = 8; saline (s.c), *n* = 4; ketamine (s.c), *n* = 8 (two-way ANOVA: saline vs ketamine, *p* = 0.01564; intranasal vs subcutaneous, *p* = 0.878); mean ± SEM]. ***E***, Representative animal location heatmaps (top) and trace (bottom) across all treatment conditions for the 1 h time point.

We found that the distance traveled by mice treated with saline is more at the 1 h time point than the distance traveled by mice administered ketamine but that there was not a significant effect of the route of delivery [Fig. 5*A*; distance traveled at 1 h: saline (i.n.), *n* = 8; ketamine (i.n.), *n* = 8; saline (s.c), *n* = 8; ketamine (s.c), *n* = 8 (two-way ANOVA: saline vs ketamine, *p* = 5.814 × 10^−5^; intranasal vs subcutaneous, *p* = 0.1261)]. Similarly, at the 24 h time point, we found that the distance traveled by mice treated with saline is more than the distance traveled by mice administered ketamine, but there was not a significant effect of the route of delivery [Fig. 5*B*; distance traveled at 24 h: saline (i.n.), *n* = 4; ketamine (i.n.), *n* = 8; saline (s.c), *n* = 4; ketamine (s.c), *n* = 8 (two-way ANOVA: saline vs ketamine, *p* = 0.03112; intranasal vs subcutaneous, *p* = 0.5562)].

Mice treated with ketamine show significantly more motionless epochs at the 1 h time point compared with those treated with saline, but there was not a significant effect of the route of delivery [Fig. 5*C*; distance traveled at 1 h: saline (i.n.), *n* = 8; ketamine (i.n.), *n* = 8; saline (s.c), *n* = 8; ketamine (s.c), *n* = 8 (two-way ANOVA: saline vs ketamine, *p* = 2.351 × 10^−4^; intranasal vs subcutaneous, *p* = 0.7992)]. Mice treated with ketamine show significantly more motionless epochs at the 24 h time point as well compared with those treated with saline, but there was not a significant effect of the route of delivery [Fig. 5*D*; distance traveled at 24 h: saline (i.n.), *n* = 4; ketamine (i.n.), *n* = 8; saline (s.c), *n* = 4; ketamine (s.c), *n* = 8 (two-way ANOVA: saline vs ketamine, *p* = 0.01564; intranasal vs subcutaneous, *p* = 0.878)].

## Discussion

We previously found that subcutaneous delivery of a single subanesthetic dose of ketamine reactivates CP-like plasticity in adult visual cortex and restores visual performance from amblyopia. This action of ketamine depends on PV+ interneuron-mediated NRG1/ErbB4 signaling and cortical disinhibition (Grieco et al., 2020, 2021). Though we have used subcutaneous delivery of ketamine in our previous studies, ketamine can be administered using several routes. Spravato (*S*-ketamine) is FDA-approved clinically for use as a nasal spray for TRD in adults with MDD and acute suicidal ideation or behavior, in conjunction with an oral antidepressant. Based on our previous work and this clinical use, we postulated that intranasal delivery of a single subanesthetic dose of ketamine would also regulate cortical disinhibition.

A major advantage of intranasal administration is that it bypasses the blood–brain barrier (BBB) and the blood–CSF barrier, avoiding side effects of systemic exposure. Intranasal administration has a main pathway of absorption into the brain, which is the olfactory routes (Erdo et al., 2018). Drug can be transferred along the olfactory nerve cells and enter the brain directly. Olfactory transfer to the brain occurs by either slow transport inside the olfactory nerve cells to the olfactory bulb or by faster transport along the extracellular space into the CSF surrounding the olfactory bulbs and the brain (Mathison et al., 1998; Illum, 2004).

**Figure 6.**
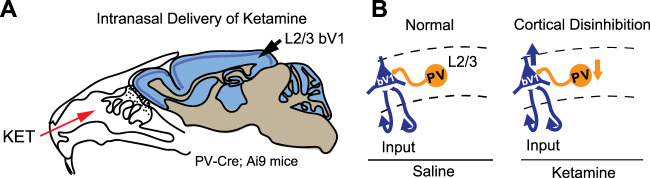
Schematic of intranasal delivery of ketamine and PV+ interneuron-mediated cortical disinhibition. ***A***, A diagram showing intranasal delivery of ketamine in mouse. ***B***, A diagram showing disinhibition in visual cortex after intranasal delivery of ketamine and the central role of PV+ interneurons (arrow-up indicates decreases in inhibition for PYRs, and arrow-down indicates decreases in excitation for PV+ interneurons).

In our present study, we use intranasal delivery of a single subanesthetic dose of ketamine and find that this results in reductions in PV+ interneuron excitation and reductions in pyramidal neuron inhibition in bV1 (Fig. 6). Intranasal ketamine decreases pyramidal neuron inhibition at the 24 and 48 h time points, and acute application of NRG1 to the bath of cortical slices rapidly increases inhibition. We found that intranasal delivery of a single subanesthetic dose of ketamine also decreases PV+ interneuron excitation and that NRG1 reverses this action of ketamine. NRG1 is a growth factor that contains the epidermal growth factor (EGF)-like domain. It plays an important role in synaptic plasticity in the nervous system and potentiates inhibitory outputs from interneurons to promote the inhibition of excitatory neurons. NRG1 stimulation of its cognate receptor tyrosine kinase ErbB4 at PV+ interneurons promote GABA release by PV+ interneurons and this inhibits the activity of pyramidal neurons in cortex, hippocampus, and amygdala (Wen et al., 2010).

Strongly supporting our hypothesis that cortical disinhibition is evoked by intranasal ketamine, we found that CREB phosphorylation at Ser133 (pCREB) increases in excitatory neurons after drug delivery. Multiple Ca^2+^-dependent signaling pathways can trigger signaling to nuclear CREB and activate CREB by phosphorylation. These pathways include a fast Ca^2+^/calmodulin (CaM)-dependent kinase (CaMK) pathway and a slower mitogen-activated protein kinase (MAPK) pathway (Cohen and Greenberg, 2008). Increased pCREB activation upregulates several immediate early genes such as c-Fos, Arc, and BDNF. The BDNF receptor TrkB can activate downstream signaling pathways such as the serine–threonine kinase mTOR, which is also involved in neural plasticity (Castrén and Monteggia, 2021). Activation of mTOR is involved in promoting ketamine-induced dendrite spine growth (Li et al., 2010; Phoumthipphavong et al., 2016; Moda-Sava et al., 2019).

In summary, intranasal delivery of a single subanesthetic dose of ketamine induces cortical disinhibition and modulates PV+ interneuron activity, and these effects are reversed by NRG1 signaling. This provides proof of principle that intranasal delivery of ketamine is effective in the mouse model and should be used in translational research to better model how ketamine is delivered to patients.

## Data Availability Statement

The data that support the findings of this study are available from the corresponding author upon reasonable request.

## Synthesis

Reviewing Editor: Matthew Grubb, King's College London Decisions are customarily a result of the Reviewing Editor and the peer reviewers coming together and discussing their recommendations until a consensus is reached. When revisions are invited, a fact-based synthesis statement explaining their decision and outlining what is needed to prepare a revision will be listed below. The following reviewer(s) agreed to reveal their identity: NONE. Note: If this manuscript was transferred from JNeurosci and a decision was made to accept the manuscript without peer review, a brief statement to this effect will instead be what is listed below.

The Reviewers differ in their opinions concerning this revision, but after consultation we agreed that addressing their remaining concerns would be a suitable basis for potential publication. Please address all of Reviewer 1's remaining issues. For Reviewer 2's outstanding concerns, please ensure you explicitly justify in the manuscript the use of only male animals. Also, please provide power analyses to justify the sample sizes employed in the behavioural experiments. Finally, for the ezTrack data please show *for each condition* a representative or average track and heat map.

Reviewer 1:

The authors have made many changes which have greatly enhanced both the accuracy and readability of the manuscript. The rearrangement of the data in figures 1,2 and 3, and the addition of the 24h timepoint in figure 4 are especially appreciated.

While the quality of the manuscript is improved, there remain a few issues.

1. The addition of wash-out data to figures 1 and 2 strengthens the point that acute NRG1 application affects IPSC/EPSC amplitude following LIV stimulation. However, this is not mentioned/discussed in the text.

2. The ratio values for the data in figure 1 (lines 143-151) are redundant as the data has moved to figure 3. It is a bit confusing to leave the values in the text.

3. The sentence: "NRG1 bath application increases EPSCs in PV interneurons" (lines 170-171) is confusing. If I understand the results correctly, this should say: "NRG1 bath application increases EPSC amplitude in ketamine-treated mice".

4. About Figure 3 and the corresponding paragraph "Sustained Cortical Disinhibition by Intranasal or Subcutaneous Delivery of Subanaesthetic Ketamine", the authors state that they observe similarity upon visual inspection. Please either perform statistics, or describe the observed effect (e.g. both intranasal and subcutaneous ketamine lead to an increase in the ratio...)

5. The data in the legend of figure 4 and supplementary table 1 do not match. For example, in the legend it states n = 5 mice for subcutaneous ketamine, but the table only shows 3 mice.

6. What is the purpose of figure 5E, and which condition is shown here. If the authors feel this information is necessary it should be shown for all conditions (saline, i.n. and s.c and ketamine i.n. and s.c.).

7. In line 107, the authors claim that ketamine modulates PV+ interneuron activity, but there is no data in the manuscript to support this claim.

8. What is the meaning of the ipsi- and contra- input in figure 6? There seems to be no distinction in the rest of the paper between different populations of pyramidal cells.

Visual abstract: It is unclear what the ipsi- and contra-input refer to.

Reviewer 2:

This article is well underpowered and the use of only males makes it even more concerning and harder to interpret. In my previous review I mentioned these points and no changes were made regarding this and the use of previously published data. Another example is when they add the ezTrack heat map and tracks (Fig 5E) and don't include what animals or conditions they come from. They should have added for each condition a representative or average track and heat map as mentioned in my review.
